# Quality-of-Life Outcomes After Transcatheter Aortic Valve Implantation in a “Real World” Population: Insights From a Prospective Canadian Database

**DOI:** 10.1016/j.cjco.2021.04.006

**Published:** 2021-04-24

**Authors:** Sandra B. Lauck, Maggie Yu, Lillian Ding, Sean Hardiman, Daniel Wong, Janarthanan Sathananthan, Jian Ye, Albert Chan, Steven Hodge, Simon Robinson, David A. Wood, John G. Webb

**Affiliations:** a St. Paul's Hospital and Vancouver General Hospital, Vancouver, British Columbia, Canada; b University of British Columbia, Vancouver, British Columbia, Canada; c Cardiac Services BC, Provincial Health Services Authority, Vancouver, British Columbia, Canada; d Royal Columbian Hospital, New Westminster, British Columbia, Canada; e Kelowna General Hospital, Kelowna, British Columbia, Canada; f Royal Jubilee Hospital, Victoria, British Columbia, Canada

## Abstract

**Background:**

Documentation of quality of life (QOL) of patients after transcatheter aortic valve implantation (TAVI) is a Canadian Cardiovascular Society quality indicator. National results have not been reported to date.

**Methods:**

We conducted an observational cohort study including all TAVI patients, irrespective of surgical risk, treated between January 2016 and June 2019 as documented in the British Columbia TAVI Registry. QOL was measured at baseline, 30 days, and 1 year, using the Kansas City Cardiomyopathy Questionnaire overall score (KCCQ-OS). We used linear regression modelling to examine factors associated with 30-day changes in QOL, logistic regression modelling to identify predictors of sustaining a poor outcome, and Cox regression modelling to ascertain risk estimates of the effect of QOL on 1-year mortality.

**Results:**

The cohort included 1706 patients (742 women [43.5%]); median age 83 years (interquartile range [IQR]: 77, 86). Median (IQR) baseline KCCQ-OS was 45 (28.2, 67), indicating severe impairment. Patients alive at 1 year (91.3%) reported a mean improvement of 24.1 (95% confidence interval [CI], 22.7-25.6) points in the KCCQ-OS at 30 days, which was sustained at 1 year (25.3; 95% CI, 23.8, 26.8). Older age, lower baseline health status, lower aortic valve gradient, lower hemoglobin, atrial fibrillation, and non-transfemoral access were associated with worse 30-day QOL. At 1 year, 65% of patients had a favorable outcome; additional risk factors for 1-year mortality (8.7%) were male sex, New York Heart Association Class IV, severe pulmonary and renal disease, diabetes, and in-patient status.

**Conclusions:**

TAVI is associated with significant early improvement in QOL, which is sustained at 1 year. The inclusion of QOL can support treatment decisions and patient-centred evaluation.

## Résumé

**Introduction:**

Les données sur la qualité de vie (QdV) des patients après l'implantation valvulaire aortique par cathéter (IVAC) sont un indicateur de qualité de la Société canadienne de cardiologie. Jusqu’à ce jour, les résultats nationaux n'ont pas été présentés.

**Méthodes:**

Nous avons mené une étude de cohorte observationnelle portant sur tous les patients ayant subi une IVAC, sans tenir compte du risque lié à l'intervention chirurgicale, qui ont été traités entre janvier 2016 et juin 2019 selon le registre d'IVAC de la Colombie-Britannique. L’évaluation de la QdV a été établie au début, 30 jours après et 1 an après au moyen du score global au Kansas City Cardiomyopathy Questionnaire (SG-KCCQ). Nous avons utilisé le modèle de régression linéaire pour examiner 30 jours après les facteurs associés aux changements dans la QdV, le modèle de régression logistique pour déterminer les prédicteurs du maintien d'issues médiocres et le modèle de régression de Cox pour établir les estimations du risque en fonction des effets de la QdV sur la mortalité 1 un après.

**Résultats:**

La cohorte regroupait 1 706 patients (742 femmes [43,5 %]); l’âge médian était de 83 ans (écart interquartile [ÉI] : 77; 86). Le SG-KCCQ médian (ÉI) au début était de 45 (28,2; 67), soit une détérioration importante. Les patients en vie un an après (91,3 %) ont signalé une amélioration moyenne de 24,1 (intervalle de confiance [IC] à 95 %, 22,7-25,6) points au SG-KCCQ 30 jours après, qui a été maintenue jusqu’à un an (25,3; IC à 95 %; 23,8; 26,8). L’âge avancé, l’état de santé initial inférieur, le gradient de la valve aortique plus faible, l'hémoglobine plus faible, la fibrillation auriculaire et l'accès non transfémoral ont été associés à une plus mauvaise qualité de vie 30 jours après. Un an après, 65 % des patients avaient des issues favorables; les facteurs additionnels de risque de mortalité un an après (8,7 %) étaient le sexe masculin, la classe IV selon la New York Heart Association, la pneumopathie et la néphropathie graves, le diabète et l’état du patient hospitalisé.

**Conclusions:**

L'IVAC est associée à une amélioration précoce significative de la QdV, qui est maintenue un an après. L'inclusion de la QdV peut contribuer aux décisions de traitement et à l’évaluation axée sur le patient.

Clinical trials and observational studies have consistently reported the health status benefits of transcatheter aortic valve implantation (TAVI) to augment the growing evidence of improved mortality, morbidity, and other clinician-reported outcomes.^1^^,^^2^ In 2016, the Canadian Cardiovascular Society (CCS) TAVI Working Group adopted the documentation of quality of life (QOL) as one of 9 quality indicators to highlight the importance of patient-reported outcomes measurements (PROMs), and strengthen the inclusion of patients’ perspectives in policy-led evaluation frameworks.^3^^,^^4^ The recently updated CCS Position Statement endorsed the measurement of QOL as a component of patient evaluation to support treatment decisions, shared decision-making, and quality monitoring of TAVI in Canada, and gave a strong recommendation to report findings stratified by sex, to account for the known difference in the pathophysiology, treatment, and outcomes between men and women with aortic stenosis.^5^^,^^6^

The British Columbia (BC) TAVI Program was implemented in 2012 to coordinate a regional system of care to leverage local expertise, accelerate access to treatment, and maximize quality of health services.^7^ The 5 BC cardiac centres provide access to transfemoral (TF) TAVI, whereas more-specialized and lower-volume procedures are primarily concentrated at a single site. The BC TAVI Registry enables regular reporting of performance indicators for the purpose of supporting local and provincial quality improvement, and guiding health policy planning. The measurement of QOL at baseline, 30 days, and 1 year after TAVI was adopted at the onset of the provincial program to strengthen the provincial evaluation framework, and conform to the CCS recommendations and the 2014 BC Ministry of Health's priority directive of shifting the culture of health care from clinician-driven to patient-centred.^8^

We report on the changes in QOL after TAVI in BC to address the current gap in evidence about health status benefits in “real-world” patients, and to contribute to current discussions about the opportunities and challenges associated with the inclusion of PROMs in the registry-based evaluation of treatment of patients with valvular heart disease.

## Methods

### Study design, data source, and population

We conducted a retrospective observational cohort study of consecutive patients who had TAVI in BC. Our study was conducted in collaboration with Cardiac Services BC (CSBC), a program of the BC Provincial Health Services Authority (BC, Canada) responsible for planning, coordinating, funding, and evaluating cardiac care across the province. The study was approved by the University of British Columbia/Providence Health Care Research Ethics Board (H18-00419).

Contribution to this registry is mandatory and is a prerequisite for provincial funding. Data documenting patient demographics, risk factors, procedural and in-hospital factors, and 30-day and 1-year follow-up were collected from the 5 BC TAVI centres. Linkages to administrative databases were conducted to validate site-reported data. The all-cause mortality rate through June 30, 2020 was obtained by linkage to the BC Vital Statistics Death Files. The analytical cohort consisted of BC patients who had a single TAVI (TF and non-TF) between January 1, 2016 and June 30, 2019.

### Health status assessment and outcomes

Patient-reported health status was assessed at baseline (pre-TAVI), 30 days, and 1 year after TAVI using the cardiac-specific 12-item Kansas City Cardiomyopathy Questionnaire (KCCQ) PROM.^9^^,^^10^ The KCCQ is a reliable instrument that has been validated in patients with aortic stenosis, is highly responsive to clinically meaningful changes, and is prognostically significant.^11^^,^^12^ The domains measured include physical limitations, symptom frequency, QOL, and social limitations. Scores range from 0 to 100, with lower scores indicating a high symptom burden and worse QOL. The subscales are combined into an Overall Summary score (KCCQ-OS), which was the primary endpoint used in this study. The secondary outcome was 1-year mortality.

### Statistical analyses

We report baseline characteristics by sex for the analytic cohort as percentages for categorical variables, and as medians with interquartile ranges (IQRs) for continuous variables. We estimated the mean scores at each time point, the mean change from baseline, and the change between time points, along with 95% confidence intervals (CIs), for the KCCQ-OS and the subscales. Changes in KCCQ scores from baseline to 30 days, and to 1 year, were evaluated using linear mixed modeling to account for the random effect of individual patients.

We employed linear regression modeling to identify predictors of change in KCCQ-OS in the first 30 days after TAVI in patients who were alive at that time point; centred mean difference of baseline score was added as an adjustment factor to account for the regression to the mean effect.^13^^,^^14^ To account for the competing effect of death, we conducted a logistic regression to examine the predictors of sustaining a poor outcome 1 year after TAVI, previously defined as experiencing at least one of either death, poor QOL (KCCQ-OS < 60), or moderate worsening in QOL (decrease of ≥ 10 points in KCCQ-OS from baseline).^15^ Finally, we used multivariable Cox regression modeling to examine the factors associated with 1-year mortality, to further ascertain the effect of baseline KCCQ-OS on survival. We explored differences in trajectories and predictors of change between men and women, to contribute to strengthening the incorporation of sex and gender into cardiovascular research.^16^

All baseline variables included in Table 1 were considered as candidate factors for multivariable analysis. A 2-sided *P* value of < 0.05 was considered statistically significant for all analyses. Parameters with a *P* value of < 0.10 were retained in the multivariable regression models. The proportional hazard assumption was confirmed for the Cox regression model. Statistical analyses were conducted using SAS (SAS Institute, Cary, NC) and R, version 4.0.2 (R Foundation, Vienna, Austria).

**Table 1:** Baseline patient characteristics by sex (N = 1706)

Characteristic	All (N = 1706)	Men (n = 964)	Women (n = 742)	*P*
Age, y	83 (77, 86)	82 (76, 86)	83 (78, 86)	**0.007**
STS risk score	3.6 (2.4, 5.4)	3.2 (2.1, 4.8)	4.1 (3.0, 6.0)	**< 0.001**
STS ≥ 8	160 (9.4)	78 (8.1)	82 (11.1)	**0.038**
Prior coronary bypass surgery	281 (16.7)	237 (25.1)	44 (6.0)	**< 0.001**
Prior coronary stenting	440 (26.2)	291 (30.9)	149 (20.2)	**< 0.001**
Prior surgical aortic valve replacement	158 (9.3)	104 (10.8)	54 (7.3)	**0.013**
Prior stroke	162 (9.6)	90 (9.5)	72 (9.8)	0.864
Atrial fibrillation	577 (34.5)	353 (37.6)	224 (30.6)	**0.003**
Prior pacemaker	189 (11.3)	120 (12.8)	69 (9.4)	**0.030**
Diabetes mellitus	494 (29.4)	290 (30.7)	204 (27.8)	0.202
LVEF < 35%	150 (8.8)	106 (11.0)	44 (5.9)	**< 0.001**
NYHA III or IV	1034 (65)	573 (64.3)	461 (66.0)	0.496
Oxygen-dependent lung disease	14 (0.8)	7 (0.7)	7 (1.0)	0.642
eGFR < 30 (mL/min)	155 (9.1)	93 (9.7)	62 (8.4)	0.354
Current dialysis	37 (2.2)	30 (3.2)	7 (1.0)	**0.002**
Body surface area, m^2^	1.9 (1.7, 2.0)	2.0 (1.8, 2.1)	1.3 (1.6, 1.9)	**< 0.001**
Hemoglobin, g/L	125 (113, 136)	128 (116, 140)	121 (110, 131)	**< 0.001**
Aortic valve area, cm^2^	0.7 (0.6, 0.9)	0.8 (0.7, 0.9)	0.7 (0.6, 0.8)	**< 0.001**
Aortic valve gradient, mm Hg	40 (32, 50)	39 (31, 47)	42 (34, 53)	**< 0.001**
Transfemoral approach	1525 (89.4)	869 (90.1)	656 (88.4)	0.249
THV device				
Balloon-expandable	1212 (71.0)	696 (72.2)	516 (69.5)	0.230
Self-expanding	425 (24.9)	219 (22.7)	206 (27.8)	0.017
Other	69 (4.0)	49 (5.1)	20 (2.7)	0.013
Outpatient at time of procedure	1480 (86.7)	819 (84.9)	661 (89.1)	**0.025**
Baseline health status				
KCCQ overall summary	45 (28.2, 67.0)	45.1 (28.4, 68.1)	44.8 (28.2, 65.1)	0.567
KCCQ physical limitations	50.0 (33.3, 75.0)	50.0 (33.3, 75.0)	50.0 (33.3, 66.7)	0.126
KCCQ symptom frequency	55.7 (37.5, 78.0)	56.3 (37.5, 79.3)	55.7 (35.5, 77.7)	0.268
KCCQ quality of life	37.5 (12.5, 50.0)	25.0 (12.5, 50.0)	37.5 (12.5, 50.0)	0.276
KCCQ social limitations	41.7 (16.7, 75.0)	41.7 (25, 75.0)	41.7 (16.7, 75.0)	0.601

Values are n (%), or median (interquartile range), unless otherwise indicated. Boldface indicates significance. STS score indicates predicted risk of operative mortality. For KCCQ, scores range from 0 to 100, with higher scores indicating less symptom burden and better quality of life.

eGFR, estimated glomerular filtration rate; KCCQ, Kansas City Cardiomyopathy Questionnaire; LVEF, left ventricular ejection fraction; NYHA, New York Heart Association; STS, Society of Thoracic Surgeons; THV, transcatheter heart valve.

### Missing data

The rate of missing KCCQ data was 14.3%, 17.3%, and 34.2% at baseline, 1 month, and 1 year, respectively. To mitigate the risks of bias associated with limiting the study to patients with complete QOL assessment that would contribute to an overestimate of favorable outcomes, (failure to complete might be due to worse health status or social determinants, for example), we chose to utilize validated strategies to maximize the data available. We employed a multiple imputation strategy to address missing baseline demographics, clinical factors, and follow-up KCCQ scores. Missing data were imputed 40 times using the multiple imputation in chained equation (MICE), under the assumption of missing at random (MAR). Sensitivity analysis for the MAR assumption is included in Supplemental Appendix S1. In the analyses of poor outcome, we limited our analyses to patients who had completed at least one QOL measurement. We examined differences in baseline characteristics between patients with a complete set of covariates and the analytical cohort, to determine the availability of a representative sample. An indicator of death was included in the multiple imputation model; however, if the patient died before completing the subsequent questionnaire, the imputed KCCQ scores were reset to missing, as patient-reported outcomes are irrelevant in the setting of death.^17^ Log and logit transformations were used to deal with non-normality. Complete data were transformed back to their original scales before analysis. Analyses run on each of the imputed dataset were pooled according to Rubin's (1987) rules ^18^ (Supplemental Appendix S1).

## Results

### Patient population

Between January 1, 2016 and June 30, 2019, a total of 1706 BC patients with severe aortic stenosis underwent TAVI in BC. Of these, 1683 (98.7%) and 1557 (91.3%) were alive at 30 days and 1 year, respectively (Supplemental Fig. S1). The baseline characteristics of the analytic cohort are summarized in Table 1. The median age was 83 years (IQR: 77, 86), and 43.5% were female. The median Society of Thoracic Surgeons (STS) predicted risk of 30-day mortality was 3.6 (IQR: 2.4, 5.4); 9.4% of patients had an STS score of ≥ 8. A total of 281 (16.7%) had had previous coronary artery bypass graft surgery; 158 (9.3%) had had previous surgical aortic valve replacement; 577 (34.5%) had atrial fibrillation; and 494 (29.4%) had diabetes. Women were more likely to be older, had higher STS scores, lower baseline hemoglobin, smaller aortic valve area, and a higher mean gradient. At the time of the procedure, 86.7% were outpatients; 89.4% had a transfemoral vascular approach. At 1 year, surviving patients who did not complete the KCCQ at all 3 time points were significantly younger (82 years [IQR 78, 86] vs 83 years [IQR 78, 86], *P* = 0.002), more likely to have had a stroke prior to TAVI (10.8% vs 7.7%, *P* = 0.036), more likely to have diabetes (31.4% vs 25.9%, *P* = 0.017), more likely to have impaired renal function (estimated glomerular filtration rate [eGFR] < 30 mL/min, 10% vs 6.7%, *P* = 0.018), and more likely to be an inpatient at the time of the procedure (81.1% vs 90.2%, *P <* 0.001). Patients with complete data at all 3 time points, and those with missing KCCQ data, did not differ significantly on their baseline KCCQ scores, except in the symptom subscale (55.3 [IQR 34.5, 75] vs 58.3 [IQR 38.7, 79.3], *P =* 0.028; Supplemental Table S1).

### Baseline health status

Median baseline KCCQ-OS was 45 (IQR 28.2, 67) points, indicating that most patients reported symptoms consistent with New York Heart Association (NYHA) Class III.^11^ In examining the subscales, the QOL domain was the most impaired (median score 37.5, IQR 12.5, 50), indicating patients’ perspective that their valve disease severely affected their overall enjoyment of life, and that they would be dissatisfied with continuing to live with the same health status. The social domain was severely impaired (median score 41.7, IQR 16.7, 75), signaling that patients’ experiences of severe aortic stenosis limited their participation in hobbies, household chores, and social interactions. Patients reported significant physical limitations (median score 50, IQR 33.3, 75), including impaired ability to walk, hurry, or attend to personal hygiene. Lastly, scores on the symptom frequency subscale (median score 55.7, IQR 37.5, 78) indicated how patients were affected by swelling, fatigue, shortness of breath, and orthopnea. There were no statistical differences in baseline QOL between men and women (Table 1).

### Temporal changes in QOL

**Figure 1:**
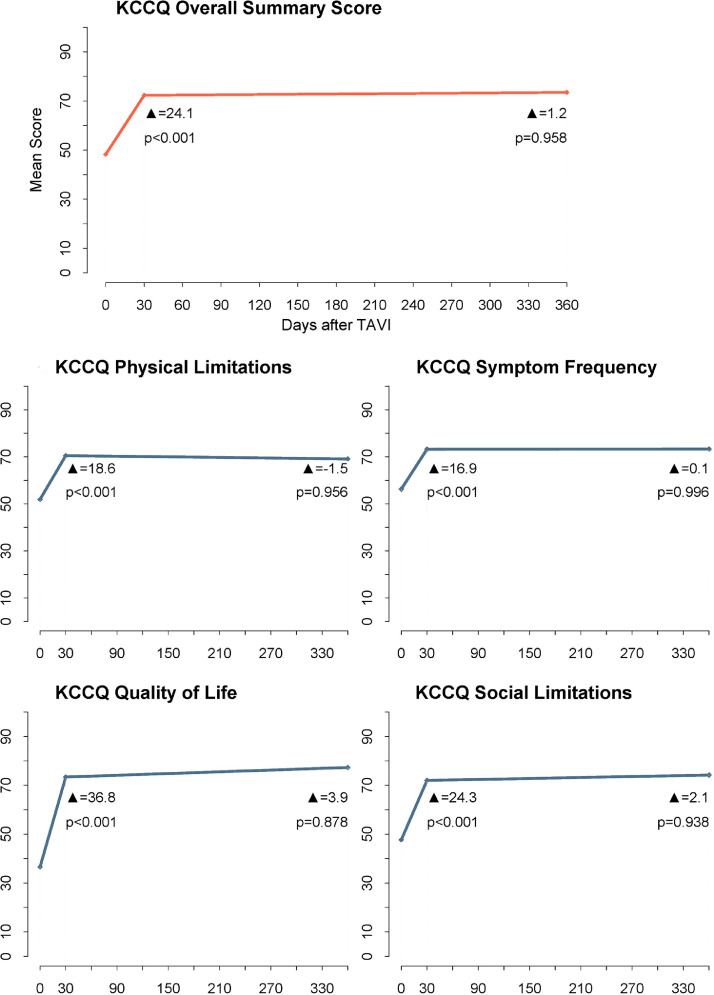
Mean changes in Kansas City Cardiomyopathy Questionnaire (KCCQ) overall summary score and subscales over time. Scores range from 0 to 100, with higher scores indicating less symptom burden and better quality of life. *P* values are for testing of the mean change in KCCQ scores between baseline and 30 days, and 30 days to 1 year. TAVI, transcatheter aortic valve implantation.

On average, participants reported a significant gain of 24.1 points on the KCCQ-OS in the first month after TAVI (*P* < 0.001), which was sustained at the 1-year time point with an adjusted mean change of 25.3 points (*P* < 0.001); a similar pattern was observed in the 4 subscales (Fig. 1; Supplemental Table S2). The adjusted estimates of changes in QOL demonstrated significant improvement from baseline to 30 days, and to 1 year (Supplemental Table S3). Both men and women experienced similar trends in improved QOL after TAVI. Although not statistically significant, women showed a more sustained improvement between the 30-day and 1-year time points (Supplemental Fig. S2).

### Factors associated with 30-day change in QOL

In the multivariable model, patients who had better health status at baseline were more likely to demonstrate significant improvement at 30 days. Every 10-point increase in baseline KCCQ-OS was associated with 2.9-point improvement in 30-day KCCQ-OS score (95% CI 2.4, 3.4; *P* < 0.001). Older age (–1 point per 5-year increase [95% CI –1.8, –0.2, *P* = 0.015], lower aortic valve gradient (–1.2 points per 10 mm Hg decrease [95% CI –0.5, –1.9, *P* = 0.002]), and atrial fibrillation (–2.5 points [95% CI –4.9, –0.1, *P* = 0.04]) were independently associated with deteriorating 30-day KCCQ-OS scores among surviving patients. In addition, the use of a non-TF vascular access approach was associated with worse health status recovery at 30 days (–5.8 points, 95% CI –9.7, –2.0, *P* = 0.003; Table 2). The models were fitted separately for men and women. In these subanalyses, prior surgical aortic valve replacement (7.7 points, 95% CI 2.2, 13.2, *P* = 0.007) was more significantly associated with improvement in KCCQ-OS for men; however, we did not observe the same association for the female patient group (Supplemental Table S4).

**Table 2:** Factors associated with change in KCCQ overall summary score between baseline and 30 days in multivariable linear regression model

Risk factor	Parameter estimate	95% CI	*P*
Age, per 5-year increase	–1.0	(–1.8, –0.2)	0.015
Male sex	2.0	(–0.3, 4.3)	0.085
Baseline KCCQ, per 10-point increase	2.9	(2.4, 3.4)	< 0.001
Aortic valve mean gradient, per 10 mm Hg	1.2	(0.5, 1.9)	0.002
Hemoglobin, per 1 g/L increase	0.1	(0.03, 0.2)	0.007
Atrial fibrillation	–2.5	(–4.9, –0.1)	0.038
Oxygen-dependent lung disease	–12.3	(–25.0, 0.4)	0.057
Outpatient	3.1	(–0.1, 6.3)	0.058
Non-transfemoral approach	–5.8	(–9.7, –2.0)	0.003

CI, confidence interval; KCCQ, Kansas City Cardiomyopathy Questionnaire.

### Factors associated with a poor outcome 1 year after TAVI

**Figure 2:**
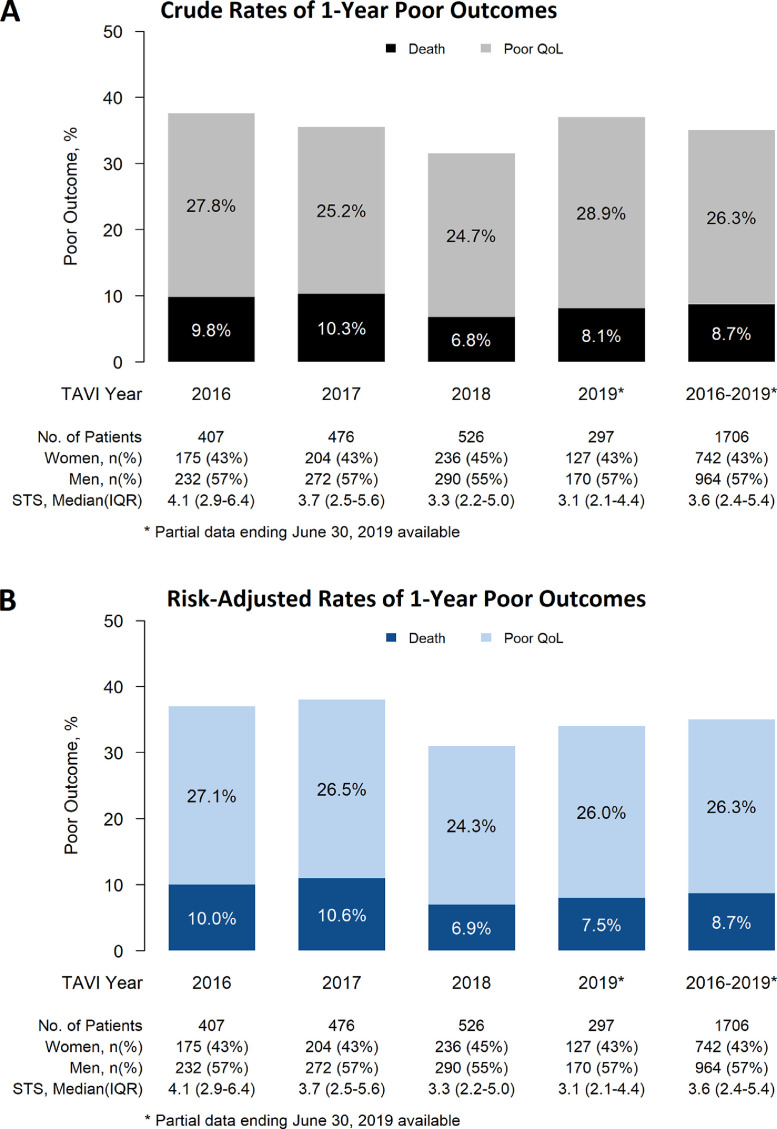
Temporal changes in (**A**) 1-year crude rates and (**B**) risk-adjusted rates of poor outcome (death and poor QoL) after TAVI (2016-2019). Rates adjusted for age, male sex, prior stroke, atrial fibrillation, aortic valve mean gradient, estimated glomerular filtration rate < 30 mL/min, baseline Kansas City Cardiomyopathy Questionnaire Overall Summary score and vascular access (transfemoral vs nontransfemoral). IQR, interquartile range; QoL, quality of life; STS, Society of Thoracic Surgeons; TAVI, transcatheter aortic valve implantation.

**Figure 3:**
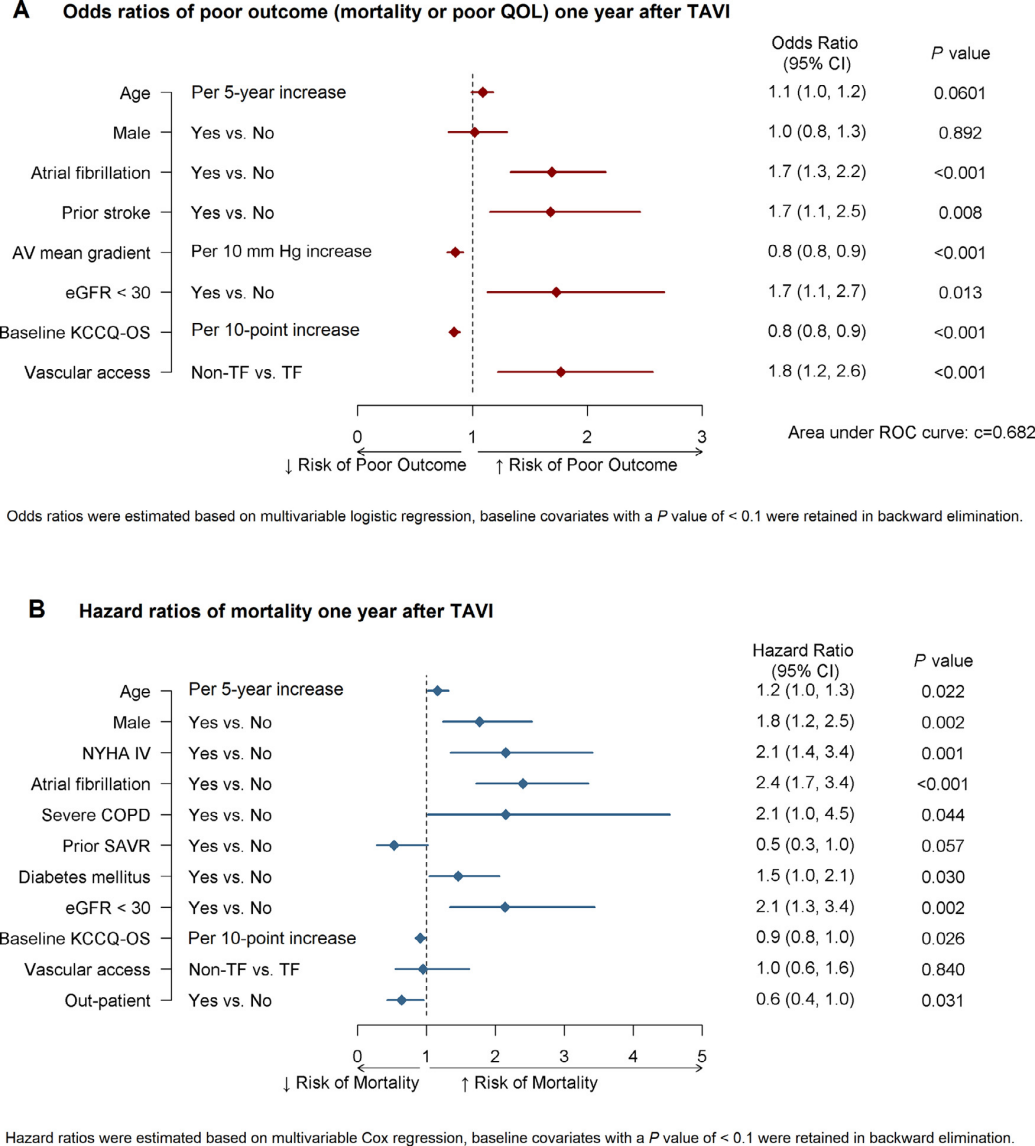
**(A)** Odds ratios of poor outcome 1 year after TAVI. Odds ratios were estimated based on multivariable logistic regression; baseline covariates with a *P* value of < 0.1 were retained in backward elimination. Poor outcome defined as sustaining at least one of either death, poor QOL (KCCQ-OS < 60), or moderate worsening in QOL (decrease of ≥ 10 points in KCCQ-OS from baseline). (**B**) Hazard ratios of mortality 1 year after TAVI. Hazard ratios were estimated based on multivariable Cox regression; baseline covariates with a *P* value of < 0.1 were retained in backward elimination. AV, aortic valve; CI, confidence interval; COPD, chronic obstructive pulmonary disorder; eGFR, estimated glomerular filtration rate; KCCQ-OS, Kansas City Cardiomyopathy Questionnaire Overall Summary Score; QoL, quality of life; NYHA, New York Heart Association; ROC, receiver operating characteristic; SAVR, surgical aortic valve replacement; TAVI, transcatheter aortic valve implantation; TF, transfemoral.

The rate of poor outcome decreased from 37.6% in 2016 to 31.5% in 2018, owing to a decrease in 1-year mortality from 9.8% to 6.8%, and a decrease in the rate of poor QOL from 27.8% to 24.7% (Fig. 2). In the multivariable logistic model, every 10-point increase in baseline KCCQ-OS score was associated with a 16% reduction of the risk of poor outcome (odds ratio [OR] 0.84, 95% CI 0.8, 0.9, *P* < 0.001). In addition, patients with preexisting atrial fibrillation (OR 1.7, 95% CI 1.3, 2.2, *P* < 0.001), previous stroke (OR 1.7, 95% CI 1.1, 2.5, *P* = 0.008), or poor renal function (eGFR < 30 mL/min; OR 1.7, 95% CI 1.1, 2.7, *P* = 0.013) were 70% more likely to derive a poor outcome 1 year after TAVI, whereas the need for a non-TF vascular access approach was associated with an 80% increase (OR 1.8, 95% CI 1.2, 2.6, *P* < 0.001) in the risk of a poor outcome (Fig. 3A).

### Factors associated with 1-year mortality

The unadjusted rate of 1-year mortality in the analytic cohort was 8.7%. In the Cox proportional hazard model, we found that every 10-point increase in the baseline KCCQ-OS score was associated with a 10% decrease in the risk of 1-year mortality (hazard ratio [HR] 0.9, 95% CI 0.8, 1, *P* = 0.026). Additional clinical characteristics that emerged as significant predictors of risk of mortality included per 5-year increase of age (HR 1.2, 95% CI 1.0, 1.3, *P* = 0.022), male sex (HR 1.8, 95% CI 1.2, 2.5, *P* = 0.002), NYHA Class IV (HR 2.1, 95% CI 1.4, 3.4, *P* = 0.001), atrial fibrillation (HR 2.4, 95% CI 1.7, 3.4, *P* < 0.001), severe pulmonary disease (HR 2.2, 95% CI 1.0, 4.5, *P* < 0.001), diabetes (HR 1.5, 95% CI 1.0, 2.1, *P =* 0.030), and severe renal disease (eGFR < 30 mL/min, HR 2.1, 95% CI 1.3, 3.4, *P* = 0.002). Those who were outpatients at the time of the procedure had a higher likelihood of 1-year survival (HR 0.6, 95% CI 0.4, 1.0, *P =* 0.031; Fig. 3B).

## Discussion

This study is the first to report changes in the health status of unselected patients undergoing TAVI in contemporary Canadian practice, using provincial registry data. We found that most patients experienced severe impairment in health status before TAVI, reported significant improvement by the 30-day time point, and sustained these QOL benefits at 1 year. Patients who did not follow this trajectory were more likely to have worse baseline health status, have lower aortic valve gradient and atrial fibrillation, be older, and require a non-TF approach. When we examined temporal changes in the composite endpoint of poor QOL and 1-year mortality, the rate of poor outcome decreased from 37.6% in 2016 to 31.5% in 2018; patients with poor health status at baseline, atrial fibrillation, stroke, or poor renal function, or who required a non-TF approach were at significantly higher risk of not achieving a good outcome. Lastly, we found that these risk factors, in addition to age, male sex, diabetes, severe pulmonary or renal disease, and urgency, accounted for a significantly higher risk of 1-year mortality.

Our findings are in keeping with the few reports of contemporary international registries available. In 2017, the STS/American College of Cardiology Transcatheter Valve Therapy (TVT) Registry reported a 27.6 and 31.9 point mean unadjusted change in KCCQ-OS at 30 days and 1 year, respectively, among patients who had TAVI in the United States between 2011 and 2016.^19^ Although these rates are numerically higher than the rates found in our study (24.1 [30-day] and 25.1 [1-year]), useful comparisons are particularly challenging in light of differences in era, with an earlier time period studied in the TVT Registry, likely in a sicker eligible population. An analysis of the EuroQoL (EQ-5D) generic health status measure employed in the German Aortic Valve Registry reported that, although health status improved in most patients, a sizable proportion failed to derive meaningful QOL benefit.^20^ Similarly, meaningful comparisons are limited because a generic health status measure is not as sensitive to detect change as a disease-specific measure such as the KCCQ, and may not appropriately capture important domains such as symptoms and function.^11^ The observed magnitude of QOL improvement was consistent with the reports of pivotal clinical trials.^21^, ^22^, ^23^, ^24^ As TAVI continues to evolve beyond the scrutiny of the early period of foundational research, the shared interest in collecting PROMs across jurisdictions suggests opportunities to align evaluation frameworks across regions to enable meaningful comparisons.

Our study further confirms the known impact of comorbid burden on patient-reported outcomes.^25^^,^^26^ We provide further evidence that patients who are not eligible for a TF vascular approach are at higher risk for worse outcomes; thus, the non-TF approach in our study population is likely a surrogate for additional comorbid burden.^27^ In addition, the findings strengthen the evidence that baseline health status is consistently found to be a powerful predictor of trajectories of change in QOL and mortality after TAVI. Multiple studies continue to demonstrate that patients who report severe impairments in their physical and social functioning, symptoms, and overall QOL at the time of their assessment are at significantly higher risk of failing to derive the survival and QOL benefits of TAVI.^28^, ^29^, ^30^ We report that approximately one-third of patients had a poor outcome, as defined by mortality or poor QOL, in keeping with existing research.^30^^,^^31^ There is strong evidence that incremental frailty, disability, and cognition are associated with trajectories of QOL after TAVI, and they are important factors to consider.^29^^,^^32^^,^^33^ To this end, the integration of PROMs into the assessment pathway offers critical information that can complement the multimodality information available to multidisciplinary teams to reach a treatment recommendation. The availability of data highlighting the predictive value of baseline health status, and the expected trajectory of change in QOL, can inform the implementation of shared decision-making and the management of patient expectations. This unique data set can be integrated into the bidirectional process to exchange information between patients and health care providers to reach a high-quality decision based on the best evidence available, and on consideration of patients’ values, priorities, and preferences.^34^, ^35^, ^36^

Our study further strengthens the evidence of differences in men's and women's clinical presentation with aortic stenosis and outcomes after TAVI. Compared with men, women are generally older, have fewer comorbidities, and have a smaller body index at the time of their procedure.^6^^,^^37^ In addition, women are 20% less likely receive treatment than men when adjusting for patient-level factors and provider impact.^38^ This context is important when examining our findings that women were significantly older, and had significantly higher surgical risk profiles in spite of lower rates of previous cardiac surgeries and interventions. Although there was not a difference in baseline QOL, men were observed to derive a more prominent improvement over time, whereas women had a higher likelihood of being alive at 1 year. Our findings augment previous research reporting the importance of parsing the effect of sex and gender on outcomes after TAVI.^39^^,^^40^

In 2012, the development of a regional system of care to facilitate access to TAVI in BC aimed to guide and monitor indications in the context of rapidly emerging evidence, provide multidisciplinary mentorship, optimize available health resources, and support excellent outcomes from the successive inception of the new provincial sites.^7^ At the time, international leaders remarked that this approach was a unique strategy to prioritize superior outcomes while promoting rational and thoughtful expanded access to care.^41^ The implementation of a centralized provincial registry was instrumental in achieving these objectives; the inclusion of PROMs to augment the evaluation framework reflected the commitment of the multidisciplinary clinical teams and policymakers to build a program aimed at improving not only the “quantity” of life afforded by the paradigm shift in the treatment of valvular heart disease, but also the quality of the years gained. Nevertheless, the integration of PROMs into clinical practice, health registries, and electronic medical records remains mostly aspirational in cardiac care.^42^ There are few examples of integrated processes that enable the use of QOL data to inform care and outcomes evaluation. In BC, the absence of measurement of QOL in the cardiac surgery program prohibits the inclusion of PROMs in the planned common evaluation framework for TAVI and surgical aortic valve replacement to shift quality reporting from being procedure-focused to disease-centred. Addressing this challenge is essential to understand differences in individual trajectories in patient-reported outcomes across treatment options that can be incorporated in future risk models. There remains a significant gap between the consensus agreement that PROMs matter, and the availability of efficient and patient-centred electronic systems to collect, analyze, and report PROMs data in a timely and effective way.^43^ Overcoming these barriers with solutions tailored to the needs of the primarily older aortic stenosis population remains a challenge across Canadian jurisdictions.^4^

### Limitations

Our study should be interpreted in light of several limitations. We highlighted the degree of missing data, and the overall challenges of collecting PROMs in clinical care. Ongoing efforts will be required by policy and clinical leaders to improve data completeness. We carefully considered the options available to account for missing health status data in our analyses. To this end, the analysis of poor outcome was limited to patients who had completed at least a single QOL measurement; we examined differences between the analytic cohort and patients for whom data were missing, and applied rigorous multiple imputations to develop a representative study cohort. In spite of these efforts, missing data should be considered as a potential source of bias in our findings.

Second, our analyses were limited by the availability of covariates in our multivariable models, and may not have fully captured patients’ risk profiles. For example, frailty, cognitive impairment, and disability are known to have deleterious effects on outcomes after TAVI;^44^^,^^45^ these important factors were not included in our models. Lastly, the 1-year QOL findings are reported in the cohort of 91.3% of patients who survived to that time; to address this challenge, we analyzed a composite endpoint of mortality and QOL to better measure the potential benefits of TAVI.

Last, although the KCCQ-OS has been validated in people with aortic stenosis, the instrument was developed to capture the experience of heart failure. In our sample, 45% of patients reported NYHA Class II or better, and more than 90% had a left ventricular ejection fraction >35%. Thus, the validity of the measurement may be limited across more complex groups, including people with fewer symptoms associated with heart failure, or other issues, including mild cognitive impairment. There is promising evidence of the availability of an instrument that addresses these limitations while strengthening the Canadian perspective on the assessment of QOL.^46^

## Conclusions

The goals of treatment of severe aortic stenosis are to extend life and enable patients to maximize their QOL. The indications for TAVI continue to evolve to achieve these patient-centred objectives. Recent advances in the way we care for patients aim at facilitating patients’ rapid recovery, and accelerating their experience of improved QOL as early as 2 weeks after TAVI, without compromising patient safety.^47^ Longitudinal studies of QOL at early and later time points, and across treatment modalities, are essential to strengthen a patient-centred approach to the treatment of valvular heart disease and inform clinical care.

## Appendix Supplementary materials

Image, application 1

## Footnotes

To access the supplementary material accompanying this article, visit *CJC Open* at https://www.cjcopen.ca/ and at doi:10.1016/j.cjco.2021.04.006.
