# Supplementary material for: Quality-of-Life Outcomes After Transcatheter Aortic Valve Implantation in a “Real World” Population: Insights From a Prospective Canadian Database
Source: CJC Open. 2021 Apr 24;3(8):1033–42. doi: 10.1016/j.cjco.2021.04.006 (PMC8413231; doi:10.1016/j.cjco.2021.04.006)

SUPPLEMENTARY MATERIAL

Quality of life outcomes after transcatheter aortic valve implantation in a "real world" population: Insights from a prospective Canadian data base

Supplemental Appendix S1: Details of multiple imputation strategy

Multiple Imputation

According to Rubin's rule, the missing at random (MAR) assumption implies that missingness is related to observed variables, but is unrelated to the missing data themselves after adjusting for all the variables in the multiple imputation model[1]. In contrast, when data are missing not at random (MNAR), missingness depends on the missing data themselves, even after controlling for other covariates (e.g., patients with poor QOL are less likely to complete QOL surveys). In our study, we incorporated baseline clinical factors, peri-procedural details, and an indicator of death that strongly predict attrition into the multiple imputation model, therefore the plausibility of the MAR assumption would increase.[2]

Sensitivity Analysis for Trend

Because it is impossible to test the MAR assumption theoretically[1], we conducted sensitivity analysis to illustrate how realistic departures from MAR would affect the results. The table below shows three scenario testing, in which we assume patients with incomplete KCCQ-OS scores had experienced small, moderate-to-large, or large-to-very large clinical changes[3]:

- Deduct 5 points from the imputed KCCQ-OS scores
- Deduct 10 points from the imputed KCCQ-OS scores
- Deduct 20 points from the imputed KCCQ-OS scores

As shown in the following table, we did not identify significant differences between observed and imputed mean KCCQ-OS scores at each time point under the three scenarios. In addition, there were no significant changes in the trend of KCCQ-OS scores between baseline and follow-up.

Sensitivity analysis scenario	Baseline	30 Days	1 Year
Observed KCCQ-OS*,<br>mean (95% CI)	49.2<br>(47.5, 50.9)	74.1<br>(72.0, 76.2)	74.7<br>(73.2, 76.3)

---

[1] Allison, P. D. (2009). *Missing data. In R. E. Millsap & A. Maydeu-Olivares (Eds.), The Sage handbook of quantitative methods in psychology (p. 72–89).* Sage Publications Ltd. https://doi.org/10.4135/9780857020994.n4

[2] Young, Rebekah, Johnson, David R. 2015. "Handling Missing Values in Longitudinal Panel Data with Multiple Imputation." Journal of Marriage and Family 77(1):277–94.

[3] John A. Spertus, Philip G. Jones, Alexander T. Sandhu, Suzanne V. Arnold, Interpreting the Kansas City Cardiomyopathy Questionnaire in Clinical Trials and Clinical Care: JACC State-of-the-Art Review, Journal of the American College of Cardiology, Volume 76, Issue 20, 2020, https://doi.org/10.1016/j.jacc.2020.09.542.

Sensitivity analysis scenario	Baseline	30 Days	1 Year
Multiply imputed KCCQ-OS under MAR assumption, mean (95% CI)	47.5 (46.3, 48.7)	71.2 (70.1, 72.3)	72.9 (71.8, 74.0)
Sensitivity analysis scenario 1: Deduct 5 points from the imputed KCCQ-OS, mean (95% CI)	46.7 (45.5, 47.9)	70.4 (69.3, 71.5)	71.8 (70.6, 72.9)
Sensitivity analysis scenario 2: Deduct 10 points from the imputed KCCQ-OS, mean (95% CI)	46 (44.8, 47.2)	69.4 (68.3, 70.5)	70.1 (68.9, 71.2)
Sensitivity analysis scenario 3: Deduct 20 points from the imputed KCCQ-OS, mean (95% CI)	44.6 (43.4, 45.8)	67.6 (66.5, 68.8)	66.7 (65.4, 67.9)

**Surviving patients with complete KCCQ-OS at baseline and follow up.*

Sensitivity Analysis for Multivariable Regression Models

To assess the impact of the assumptions made about missing data on the multivariable modeling, we conducted regression analyses again using datasets with complete covariates (i.e., complete-case analysis). The intent of performing sensitivity analysis is not to demonstrate that any given model is correct, it is simply to illustrate the departures of modeling results between the MAR and the MNAR assumptions.

1. Factors associated with 30-day change in KCCQ-OS

The table below includes parameter estimates (95% CI) based on the multivariable linear regression modeling for factors associated with 30-day change in KCCQ-OS using complete data. In comparison to the original model results, moderate or severe aortic valve regurgitation was entered into the multivariable model as an additional factor significantly associated with 30-day change in KCCQ-OS. We did not find significant changes in the directions of association for other risk factors retained in the model.

Risk Factor	Parameter Estimate (95% CI)	*P* value
Age, per 5 years increase	-0.2 (-0.4, -0.1)	0.004
Male sex	2.4 (0.1, 4.8)	0.044
Baseline KCCQ, per 10 points increase	3.3 (2.8, 3.8)	<0.001
AV mean gradient, per 10 mm Hg	1.2 (0.4, 2.0)	0.002
Hemoglobin, per 1 g/L increase	0.1 (0.04, 0.2)	0.002
Atrial fibrillation	-2.5 (-5.0, -0.03)	0.048
Oxygen-dependent lung disease	-14.8 (-27.8, -1.9)	0.025
eGFR < 30 ml/min	-4.8 (-9.7, 0.2)	0.059
Moderate or severe aortic valve regurgitation	3.9 (0.6, 7.1)	0.020
Out-patient at time of procedure	1.8 (-1.9, 5.6)	0.333

Risk Factor	Parameter Estimate (95% CI)	*P* value
Non-Transfemoral approach	-6.5 (-10.6, -2.4)	0.002

2. Factors associated with 1-year poor outcome

We re-fit the multivariable logistic regression model for complete case analysis. In this model, factors strongly associated with mortality, such as prior SAVR and NYHA IV, emerged as significant predictors of 1-year poor outcome; most of the factors from the original multivariable model were retained here.

Risk Factor	Odds Ratio (95% CI)	*P* value
Age, per 5 years increase	1.1 (0.9, 1.2)	0.377
Male, Yes vs. No	1.1 (0.8, 1.5)	0.720
Atrial fibrillation, Yes vs. No	2.2 (1.6, 3.0)	< 0.001
Prior SAVR, Yes vs. No	0.4 (0.2, 0.7)	0.002
NYHA IV, Yes vs. No	2.3 (1.3, 4.0)	0.003
AV mean gradient, per 10 mm Hg increase	0.8 (0.7, 0.9)	< 0.001
eGFR < 30, Yes vs. No	2.0 (1.1, 3.6)	0.015
Baseline KCCQ-OS, per 10 points increase	0.8 (0.7, 0.8)	< 0.001

3. Factors associated with 1-year mortality

In comparison to the multivariable Cox regression model using imputed data, prior pacemaker was added as a marginally significant predictor of 1-year mortality (HR 1.6, 95% CI 1.0, 2.6; p=0.064). There were no changes in the direction of association for other risk factors which were included in the original model.

Risk Factor	Hazard Ratio (95% CI)	*P* value
Age, per 5 years increase	1.2 (1.0, 1.4)	0.025
Male, Yes vs. No	1.6 (1.0, 2.5)	0.032
NYHA IV, Yes vs. No	2.0 (1.1, 3.5)	0.015
Atrial fibrillation, Yes vs. No	2.3 (1.6, 3.5)	< 0.001
Severe COPD, Yes vs. No	2.7 (1.2, 6.1)	0.015
Prior SAVR, Yes vs. No	0.5 (0.2, 1.2)	0.099
Prior pacemaker, Yes vs. No	1.6 (1.0, 2.6)	0.064
Diabetes mellitus, Yes vs. No	1.7 (1.1, 2.6)	0.012
eGFR < 30, Yes vs. No	2.3 (1.3, 4.0)	0.003
Baseline KCCQ-OS, per 10 points increase	0.9 (0.8, 1.0)	0.002
Vascular access: Non-Transfemoral vs. Transfemoral	0.7 (0.4, 1.6)	0.442
Out-patient, Yes vs. No	0.6 (0.4, 1.0)	0.038

Supplemental Table S1: Baseline Characteristics of Patients with Complete and Missing KCCQ Measurements

Patient Characteristics	Completed all KCCQ (N = 819)	Missing KCCQ (N = 738)	*P* value
Age (years) median (IQR)	83 (78, 86)	82 (77, 86)	**0.002**
Female	362 (44.2%)	336 (45.5%)	0.599
STS score (%) median (IQR)	3.4 (2.3, 5.1)	3.7 (2.5, 5.6)	0.051
STS > 8%	62 (7.6%)	67 (9.1%)	0.277
Prior coronary bypass surgery	134 (16.5%)	119 (16.6%)	0.971
Prior coronary stenting	219 (26.9%)	174 (24.2%)	0.232
Prior surgical aortic valve replacement	68 (8.3%)	75 (10.2%)	0.205
Prior stroke	63 (7.7%)	78 (10.8%)	**0.036**
Atrial fibrillation	249 (30.8%)	247 (34.4%)	0.131
Prior pacemaker	86 (10.6%)	81 (11.3%)	0.640
Diabetes mellitus	211 (25.9%)	226 (31.4%)	**0.017**
LVEF < 35%	56 (6.8%)	71 (9.6%)	0.045
NYHA III or IV	511 (65.8%)	429 (63.5%)	0.359
Oxygen-dependent lung disease	8 (1%)	4 (0.6%)	0.346
eGFR<30 (mL/min)	55 (6.7%)	74 (10%)	**0.018**
Current dialysis	8 (1%)	22 (3.1%)	0.004
Body surface area (m^2) median (IQR)	1.9 (1.7, 2.0)	1.9 (1.7, 2.1)	0.477
Hemoglobin (g/L) median (IQR)	127 (115, 136)	125 (112, 137)	0.126
Aortic valve area (cm^2) median (IQR)	0.7 (0.6, 0.9)	0.7 (0.6, 0.9)	**0.023**
Aortic valve gradient (mm Hg) median (IQR)	41 (33, 50)	40 (32, 50)	0.183
Transfemoral approach	738 (90.1%)	654 (88.6%)	0.340
THV device:			
Balloon-expandable	603 (73.6%)	501 (67.9%)	0.013
Self-expanding	175 (21.4%)	216 (29.3%)	0.0003
Other	41 (5%)	20 (2.7%)	0.020
Out-patient at time of procedure	739 (90.2%)	598 (81.1%)	**<0.001**
Baseline Health Status			

**Patient Characteristics**	**Completed all KCCQ (N = 819)**	**Missing KCCQ (N = 738)**	***P* value**
KCCQ overall summary median (IQR)	46.9 (29.9, 68.8)	44.5 (27.5, 65.9)	0.109
KCCQ physical limitations median (IQR)	50.0 (33.3, 75.0)	50.0 (33.3, 75.0)	0.114
KCCQ symptom frequency median (IQR)	58.3 (38.7, 79.3)	55.3 (34.5, 75.0)	**0.028**
KCCQ quality of life median (IQR))	37.5 (12.5, 62.5)	37.5 (12.5, 50.0)	0.438
KCCQ social limitations median (IQR)	41.7 (25.0, 75.0)	41.7 (16.7, 75.0)	0.586

Supplemental Table S2: Unadjusted mean quality of life scores and changes from baseline

	**Baseline** Mean (95% CI)	**30-Day** Mean (95% CI)	**1-Year** Mean (95% CI)	**Change from baseline to 30 days** Mean (95% CI)	**Change from baseline to 1 year** Mean (95% CI)
**KCCQ Overall Summary Score**	48.1 (47.1, 49.1)	72.1 (71.1, 74.1)	73.1 (72.1, 75.1)	24.1 (23.1, 26.1)	25.1 (24.1, 27.1)
**Physical Limitations**	52.1 (51.1, 53.1)	71.1 (69.1, 72.1)	69.1 (68.1, 71.1)	19.1 (17.1, 20.1)	17.1 (15.1, 19.1)
**Total Symptoms**	56.1 (55.1, 58.1)	73.1 (72.1, 75.1)	73.1 (72.1, 75.1)	16.9 (15.3, 18.5)	17.1 (15.1, 19.1)
**Quality of Life**	37.1 (35.1, 38.1)	73.1 (72.1, 75.1)	77.1 (76.1, 79.1)	37.1 (35.1, 39.1)	41.1 (39.1, 42.1)
**Social Limitations**	48.1 (46.1, 49.1)	72.1 (70.1, 74.1)	74.1 (73.1, 76.1)	24.1 (22.1, 26.1)	26.1 (25.1, 28.1)

Supplemental Table S3: Adjusted mean quality of life scores and changes from baseline

QOL Scale	Time Point	Adjusted Mean (95% CI) *	Time Interval	Adjusted Change from Baseline (95% CI) *	*p*-Value*
**KCCQ Overall Summary**	30 days	72.3 (71.1-73.4)	Baseline to 30 days	24.1 (22.7-25.6)	<.0001
	1 year	73.5 (72.2-74.7)	Baseline to 1 year	25.3 (23.8-26.8)	<.0001
**KCCQ Physical Limitations**	30 days	70.5 (69.2-71.8)	Baseline to 30 days	18.6 (17.0-20.2)	<.0001
	1 year	69.1 (67.6-70.5)	Baseline to 1 year	17.1 (15.4-18.9)	<.0001
**KCCQ Symptom**	30 days	73.2 (72.0-74.4)	Baseline to 30 days	16.9 (15.3-18.5)	<.0001
	1 year	73.3 (72.0-74.6)	Baseline to 1 year	17.0 (15.3-18.7)	<.0001
**KCCQ Quality of Life**	30 days	73.4 (72.0-74.7)	Baseline to 30 days	36.7 (35.0-38.5)	<.0001
	1 year	77.3 (75.9-78.7)	Baseline to 1 year	40.7 (38.8-42.5)	<.0001
**KCCQ Social Limitations**	30 days	72.0 (70.6-73.5)	Baseline to 30 days	24.3 (22.4-26.2)	<.0001
	1 year	74.2 (72.7-75.7)	Baseline to 1 year	26.5 (24.6-28.4)	<.0001

*Adjusted values, confidence intervals, and *p*-values based on mixed effects models

Supplemental Table S4: Factors associated with change in KCCQ-OS between baseline and 30 days in multivariable linear regression model, by sex

**Risk Factor**	**Parameter Estimate**	**95% CI**	***P*- value**
Men			
Age, per 5 years increase	-0.7	(-1.7, 0.3)	0.188
Baseline KCCQ-OS, per 10 points increase	3.0	(2.3, 3.6)	<0.001
Non-Transfemoral approach	-5.7	(-10.9, -0.6)	0.029
AV mean gradient, per 10 mm Hg increase	1.5	(0.4, 2.2)	0.005
Hemoglobin, per 1 g/L increase	0.1	(0.03, 0.2)	0.01
Out-patients	4.9	(0.7, 9.0)	0.021
**Prior surgical aortic valve replacement**	7.7	(2.2, 13.2)	**0.007**
Women			
Age, per 5 years increase	-0.9	(-2.1, 0.3)	0.136
Baseline KCCQ-OS, per 10 points increase	2.9	(2.2, 3.7)	<.0001
Non-Transfemoral approach	-6.4	(-12.1, -0.7)	0.028
AV mean gradient, per 10 mm Hg increase	1.2	(0.1, 2.2)	0.029
Hemoglobin, per 1 g/L increase	0.1	(-0.002, 0.2)	0.055
Atrial fibrillation	-4.0	(-7.8, -0.2)	0.037

Supplemental Figure S1: Flowchart of the Analytic Cohorts

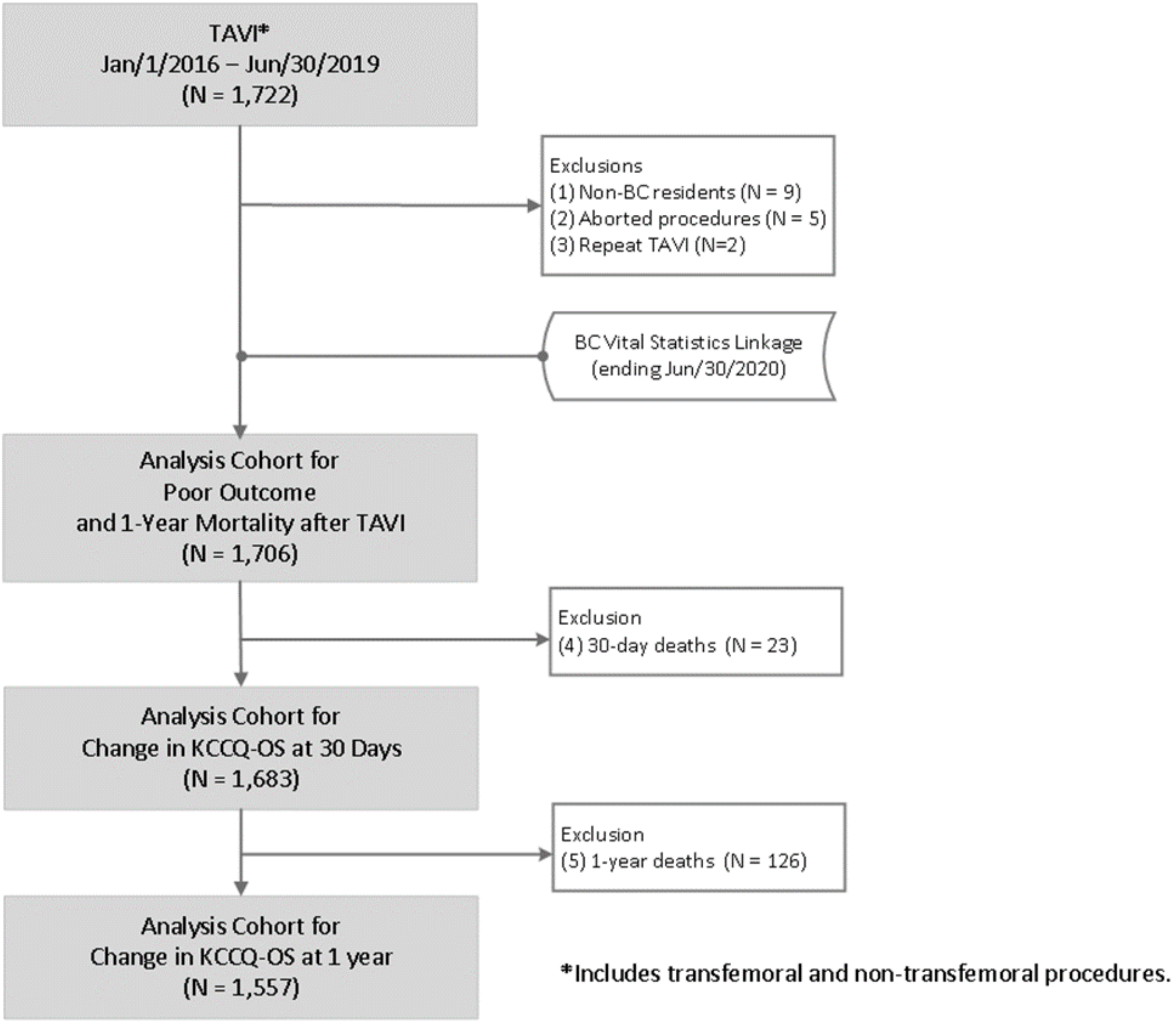

Supplemental Figure S2: Mean changes in Kansas City Cardiomyopathy Questionnaire overall summary score (KCCQ-OS) and sub-scales over time by sex
Scores range from 0 to 100, with higher scores indicating less symptom burden and better quality of life

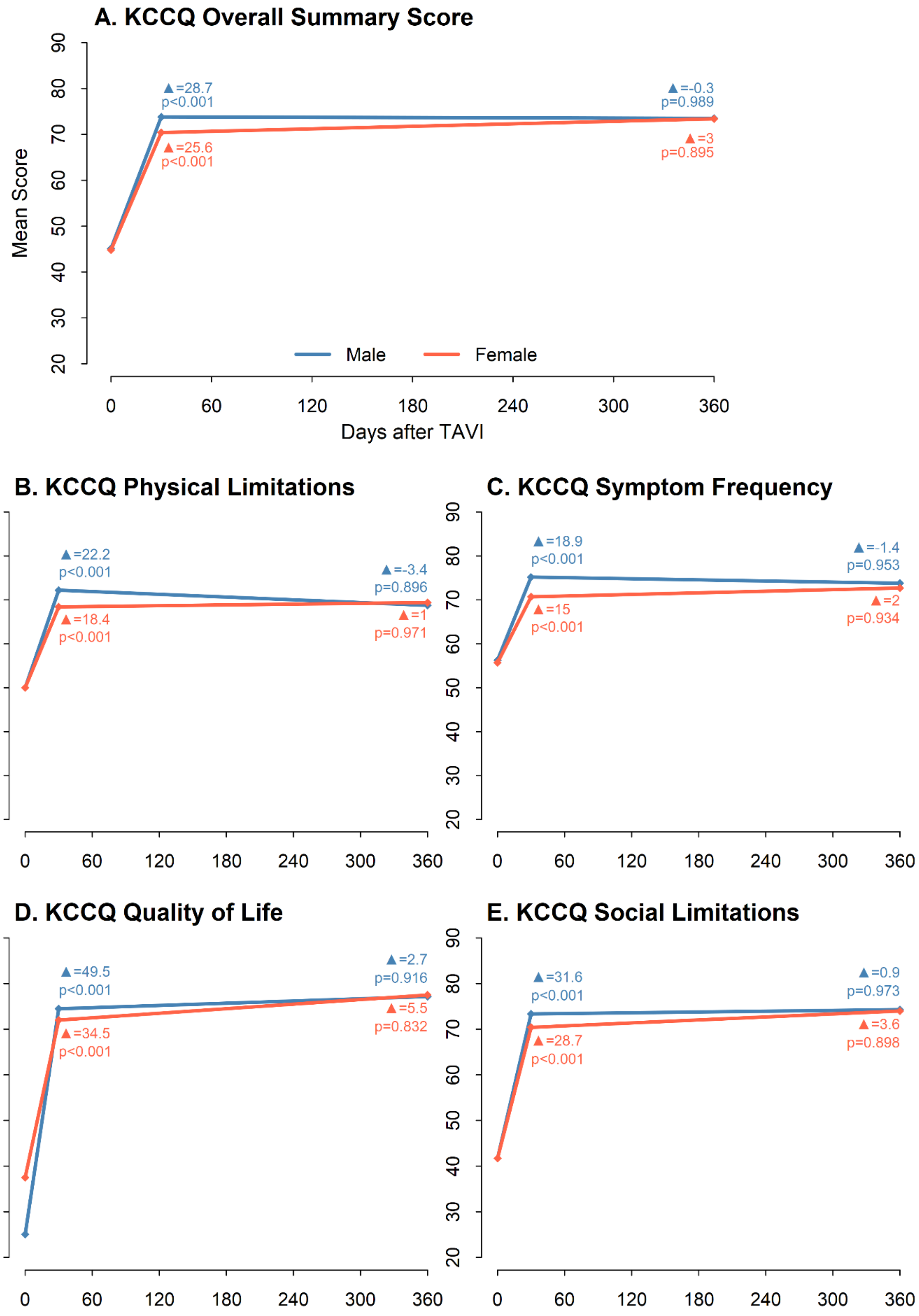

Supplement: Supplementary file 1 [file mmc1.pdf]
